# A systematic review of cognitive telerehabilitation in patients with cognitive dysfunction

**DOI:** 10.3389/fneur.2024.1450977

**Published:** 2025-01-15

**Authors:** Hyeonwoo Jeon, Doo Young Kim, Si-Woon Park, Bum-Suk Lee, Hyeong-Wook Han, Namo Jeon, Minsong Kim, Mingu Kang, Suebeen Kim

**Affiliations:** ^1^Department of Rehabilitation Medicine, International St. Mary's Hospital, Catholic Kwandong University College of Medicine, Incheon, Republic of Korea; ^2^International St. Mary's Hospital, Catholic Kwandong University College of Medicine, Incheon, Republic of Korea

**Keywords:** telerehabilitation, cognitive remediation, cognitive dysfunction, neurodegenerative diseases, health services accessibility

## Abstract

**Introduction:**

One of the possible treatment options for patient with cognitive dysfunction is cognitive telerehabilitation. Previous systematic reviews on cognitive telerehabilitation have focused on specific disease groups and the analysis of intervention methods did not differentiate between traditional face-to-face cognition treatment and usual care. In this systematic review, we aim to analyze randomized controlled trials (RCTs) that compare telerehabilitation with face-to-face treatment or usual care for improving cognitive function in elderly individuals with cognitive dysfunction or patients with acquired brain injury.

**Methods:**

We conducted this systematic review following the guidelines of the Preferred Reporting Items for Systematic Reviews and Meta-Analyses (PRISMA). In this systematic review, we searched 7 electronic databases (PubMed, Cochrane, EMbase, CINAHL, Web of Science, Scopus, KMbase) to identify relevant studies published through December 10, 2024. We conducted a meta-analysis to assess the quality of the studies and synthesize the evidence. Certainty of evidence was evaluated using the Grading of Recommendations, Assessment, Development, and Evaluation (GRADE) method.

**Results:**

Finally, 16 studies were included in the analysis. For comparing telerehabilitation with face-to-face cognition treatment, the meta-analysis included 2 RCTs for global cognition (immediate outcome), 2 RCTs for attention (immediate outcome), 2 RCTs for visuospatial function (immediate outcome). For comparing telerehabilitation with usual care, the meta-analysis included 7 RCTs for global cognition (immediate outcome), 3 RCTs for global cognition (persistence outcome), 4 RCTs for attention (immediate outcome), 3 RCTs for executive function (immediate outcome), 3 RCTs for working memory (immediate outcome), 3 RCTs for visuospatial function (immediate outcome).

**Discussion:**

Telerehabilitation has been shown to be more effective than usual care in improving global cognitive function, and its effectiveness is not inferior to that of traditional face-to-face cognitive treatment. By overcoming the limitations of traditional cognition rehabilitation and providing continuous treatment, telerehabilitation can offer effective treatment in specific situations.

## Introduction

Cognitive dysfunction is the result of age-related neurodegenerative changes (1). It is also a major complication in acquired brain injury, such as stroke, or traumatic brain injury (TBI) (2, 3). Cognitive dysfunction interferes with functional abilities and activities of daily living (ADLs), thereby reducing people’s quality of life (QOL) and participation in society (4). Mild cognitive impairment (MCI) occurs in 15–20% of elderly, and it is known that 8–15% of those with MCI progress to dementia each year (5). Cognitive dysfunction is reported to occur in 70% of overall stroke survivors, 15% of mild TBI patients, and 65% of moderate-to-severe TBI patients (2, 3).

Cognitive rehabilitation interventions for patients with cognitive dysfunction is essential, with a particular emphasis on early implementation to preserve and enhance individual’s independence in ADLs (6). Cognitive training has also been recognized as an effective intervention strategy for improving or preserving cognitive function in patients with cognitive dysfunction (1). It can address both physiological and pathological neurodegenerative processes by stimulating the brain’s compensatory mechanisms (1). Therefore, appropriately designed cognitive training programs can effectively activate the neural systems involved in sensory and cognitive processing and take advantage of the brain’s plasticity to restore brain and cognitive function to a normal state (7).

However, traditional face-to-face cognition treatment approaches have the disadvantage of accessibility issues (8). Barriers to treatment involve restricted service accessibility, especially post-transition from hospital to home, limited mobility due to physical and cognitive impairments, and reduced levels of participation in the face-to-face cognition rehabilitation programs (9, 10). These accessibility issues interrupt the continuity of treatment. These issues have been exacerbated by the COVID-19 pandemic, underscoring the need for additional training options for maintaining continuity of rehabilitation (1). Telerehabilitation has emerged as a promising approach to address numerous challenges, including dependence on caregivers, financial constraints, insufficient access to local medical resources, and transportation difficulties and has shown high participant satisfaction (10, 11). In the field of cognitive treatment, telerehabilitation is emerging as a promising treatment alternative to traditional face-to-face cognition rehabilitation, through technological advances (12). Telerehabilitation is defined by the American Telemedicine Association as the delivery of rehabilitation services using information and communication technologies, and utilizes telecommunications, remote sensing, and operational technology to deliver medical rehabilitation services remotely (6, 13). This approach improves accessibility, providing more effective treatment opportunities, allowing treatment to continue despite spatial constraints (14).

To the best of our knowledge, previous systematic reviews on cognitive telerehabilitation have focused on specific disease groups and the analysis of intervention methods did not differentiate between traditional face-to-face cognition treatment and usual care. The aim of this systematic review is to analyze and synthesize evidence on the efficacy of cognitive telerehabilitation treatment in patients with cognitive dysfunction and compare it to conventional face-to-face cognition treatment group or usual care group.

## Materials and methods

### Review question

Does cognitive telerehabilitation improve cognitive function (attention, memory, visuospatial, executive function), activities of daily living, quality of life in patients with cognitive dysfunction?

This literature review aims to assess studies of various forms of telerehabilitation in patients with cognitive dysfunction.

### Registration of the study protocol

We conducted this systematic review following the guidelines of the Preferred Reporting Items for Systematic Reviews and Meta-Analyses (PRISMA) and flow diagram. The protocol of this review was registered in the International Prospective Register of systematic reviews (PROSPERO) under the following registration number CRD 42023454250 and can be accessed in its entirety on the program website.^1^

### Criteria for this review (PICO)

1. Patients (P): Patients with cognitive dysfunction (stroke, traumatic brain injury, neurodegenerative diseases, cognitive dysfunction).
2. Intervention (I): Telerehabilitation.
3. Comparison (C): Face-to-face cognition treatment or Usual care.
4. Outcomes (O): Cognition (memory, attention, executive function, visuospatial function), activities of daily living (ADLs), quality of life (QOL).

Usual care was defined as receiving no treatment, sham treatment, etc., while the face-to-face treatment was defined as receiving traditional therapy provided directly by a therapist. Further details are presented in Table 1.

**Table 1:** Characteristics of included studies.

1^st^ author	Title	Journal	Year	Design	Intervention	Comparison	Assessment	Outcome tool	Outcomes	Remark
Calabrò (19)	Benefits of telerehabilitation for patients with severe acquired brain injury: promising results from a multicenter randomized controlled trial using nonimmersive virtual reality	Journal of Medical Internet Research	2023	RCT	Teleneuro-Virtual Reality Rehabilitation System (VRRS HomeKit device): 1 h/session, 5 sessions/week for 12 weeks	[face-to-face treatment group] Usual Territorial Rehabilitative Treatment (paper and pencil in a face-to-face rehabilitative setting): 1 h/session, 5 sessions/week for 12 weeks	T0: Baseline T1: Post-treatment examinations (week 12)	[general cognitive function] MoCA [executive function] FAB [ADLs] BI [QoL] SF-36	Both teleneuro-VRRS and FTF groups improved in global functional, cognitive, and general health status. However, Only the teleneuro-VRRS group improved in executive functions, with a significant reduction in anxiety and depression symptoms. The teleneuro-VRRS group achieved a statistically significant improvement: general and motor outcomes, psychological well-being, QoL.	Best improvement: BI (*p* < 0.001), FAB (*p* < 0.001), BDI-II (*p* < 0.001) Burden of caregivers (CBI; *p* < 0.004) Statistical differences (between-group analysis): anxiety (effect size [ES] = 0.85, *p* < 0.02), self-control (ES = 0.40, *p* < 0.03) subtests of the PGWBI and in the social role functioning (ES = 0.85, *p* < 0.02) subtest of the SF-36, confirmed by quite medium and large ESs.
Canyazo (22)	Effectiveness of cognitive rehabilitation on mild cognitive impairment using teleneuropsychology	Dementia & Neuropsychologia	2023	RCT	AgeWise program (Computerized cognitive rehabiliation program): 45 min/session, 1 session/week for 10 weeks	[Usual care group] (No treatment (Waiting list))	Baseline Post-treatment (week 10)	[general cognitive function] MoCA [verbal memory] RAVLT EDO-10 MMQ [depression] GDS [anxiety] DASS-21 NPI-Q FAQ	Treatment group (week 10) had better scores in cognitive variables. Memory (RAVLT learning trials *β* = 0.7; *p* = 0.030) RAVLT delayed recall (*β* = 0.48; *p* = 0.029) Activities of daily living (FAQ *β* = −3.16; *p* = 0.001) Satisfaction with memory performance (MMQ satisfaction *β* = 10.3; *p* = 0.004) Use of memory strategies (MMQ strategy *β* = 4.4; *p* = 0.00)	Significant reduction of affective symptomatology: depression (GDS *β* = −2.68; *p* = 0.00), neuropsychiatric symptoms (NPI-Q *β* = −1.46; *p* = 0.045), forgetfulness (EDO-10 *β* = −1.5; *p* = 0.00), and stress (DAS stress *β* = −6.0; *p* = 0.00)
Charvet (15)	Cognitive function in multiple sclerosis improves with telerehabilitation: Results from a randomized controlled trial	PLOS ONE	2017	RCT	Adaptive cognitive remediation (ACR) program (Telerehabilitation): 1 h/session, 5 sessions/week for 12 weeks	[Usual care group] ordinary computer games: 1 h/session, 5 sessions/week for 12 weeks	Baseline Post-treatment (week 12)	[general cognitive function] Neuropsychological Composite Score [Working memory] WAIS-IV Letter Number Sequence [Working memory] WAIS-IV DSB [Processing speed] Paced Auditory Serial Addition Test [Verbal learning] SRT [Visual learning] BVMT-R [Visual scanning] Delis-Kaplan Executive Function System Trails	Participants in the ACR had significantly greater improvement in the primary outcome of cognitive functioning (z score ± SD: 0.25 ± 0.45 vs. 0.09 ± 0.37) At study end, more active condition participants (56.7% vs. 31.0%) reported experiencing an improvement in cognition over the 12-week duration of the study. (indicating a rating of 1.0 for improved, versus no change of 0.0, and − 1.0 for decline: ACR vs. active control, mean ± SD = 0.52 ± 0.59 vs. 0.28 ± 0.52, *p* = 0.007)	Data unavailable.
Jelcic (20)	Feasibility and efficacy of cognitive telerehabilitation in early Alzheimer’s disease: a pilot study	Clinical Interventions in Aging	2014	RCT	Lexical-Semantic Stimulation with teleconference technology (LSS-Tele): 1 h/session, 2 sessions/week for 3 months	[face to face treatment group] direct: 1 h/session, 2 sessions/week for 3 months	Baseline Post-treatment (month 3)	[general cognitive function] MMSE [attention] Digit Cancelation Test Trail making test A [Executive] Trail making test B [working memory] DSF DSB [visual spatial memory] ROCF Copy Test [verbal memory] RAVLT [visual memory] ROCF Delayed Recall Test Brief Story Recall	Mean MMSE score: improved significantly in LSS-tele and LSS-direct treatments. LSS-tele improved language abilities (phonemic, semantic), stabilized delayed verbal episodic memory (improved performance after the LSS-direct intervention). For episodic memory, delayed verbal memory stabilized after LSS-tele and improved only after LSS-direct intervention, with respect to deterioration in the control group. Immediate episodic memory (story immediate recall) improved significantly only in the LSS-direct group (*p* = 0.03). Attention abilities assessed with the Digit Cancellation Test improved significantly only in the LSS-tele group (*p* = 0.01).	The results of comparing telerehabilitation group and face-to-face treatment group, telerehabilitation group and usual care group were used for meta-analysis, respectively.
Jelcic (20)						[Usual care group] Unstructured cognitive stimulation			LSS-tele improved stabilized delayed verbal episodic memory (improved performance after the LSS-direct intervention, verbal episodic memory decline observed in the usual care group). Improvement was not achieved in any neuropsychological test score after unstructured cognitive stimulation.	
Jonsdottir (23)	Virtual reality for motor and cognitive rehabilitation from clinic to home: a pilot feasibility and efficacy study for persons with chronic stroke	Frontiers in Neurology	2021	RCT	Phase I: Clinic HEAD 45 min/session, 3 sessions/week for 4 weeks Phase II: Home HEAD (tele-VRRS): 45 min/session, 3 sessions/week for 3 months Usual care for 3 months	[Usual care group] Phase I: Clinic HEAD 45 min/session, 3 sessions/week for 4 weeks Phase II: Usual care for 6 months	T0: Baseline T1: End of the Clinic HEAD T2: 3 months after T3: At follow up 7 months after baseline	[general cognitive function] MoCA (Montreal Cognitive Assessment) [Memory] RBMT-GMI	Clinic HEAD result: significant increase in cognition (*p* = 0.003), most secondary outcome variables There was an improvement in memory at 6 months from Clinic HEAD only in the Home HEAD group, indicating further long-term benefit on memory from bringing the HEAD system home.	The Human Empowerment Aging and Disability program (HEAD) protocol was feasible with good adherence: Clinic HEAD phase (92%), Home HEAD phase (89%)
Koc (24)	Comparison of the effect of online physical exercise and computerized cognitive stimulation in patients with Alzheimer’s disease during the Covid-19 pandemic	Complementary Therapies in Clinical Practice	2024	RCT	Online supervised physical exercise program (SPEP) : 60 min/session 2 sessions/ week for 12 weeks Cognitive stimulation (CS) program : 10 min/session at least 3 ~ 5 days for 12 weeks	[Usual care group] : No treatment	T1 (Baseline) T2 (12 weeks) T3 (24 weeks)	[general cognitive function] MoCA [QoL] ADRQL [ADLs] Katz ADL scale, Lawton IADL scale	For cognition intragroup outcomes, the usual care group significantly reduced their MoCA during the study process. Physcial exercise + Cognitive stimulation group demonstrated significant improvement in cognition, balance and reduction in depression compared to the Control group (*p* < 0.05).	
Mahncke (7)	A randomized clinical trial of plasticity-based cognitive training in mild traumatic brain injury	Brain	2021	RCT	BrainHQ, Posit Science (telerehabilitation) experimental treatment: 1 h/session, 5 sessions/week, for 13 weeks	[Usual care group] Sham_games: 13 off-the-shelf computer games (e.g., hangman, Boggle, mah-jong) similar to the experimental treatment program: 1 h/session, 5 sessions/week for 13 weeks	V1: Baseline V2: After training (week 13) V3: No-training follow-up period (month 3)	[general cognitive function] Nine well-standardized measures (1 ~ 5 RAVLT +2 ~ 10 RULIT + WAI + WMS + EXAMINER battery) [general cognitive function] CFQ [executive function] FrSBe [depression] BDI [QoL] SF-12 (Short-Form 12 Physical/Mental Component Score) [ADLs] TIADL (Timed Instrumental Activities of Daily Living)	Telerehabilitation group showed an composite cognitive measure improvement: the post-training [+6.9 points, confidence interval (CI) + 1.0 to +12.7, *p* = 0.025, d = 0.555], follow-up visit (+7.4 points, CI + 0.6 to +14.3, *p* = 0.039, *d* = 0.591) Both large and small cognitive function improvements were seen twice as frequently in the treatment group. Statistically equivalent improvements in both groups: depressive and cognitive symptoms.	No significant between group effects were seen on other measures. (directly observed functional TIADL and symptom measures) Data unavailable.
Manenti (12)	Effectiveness of an innovative cognitive treatment and telerehabilitation on subjects with mild cognitive impairment: a multicenter, randomized, active-controlled study	Frontiers in Aging Neuroscience	2020		Face-to-face Virtual reality rehabilitation system (clinic-VRRS: 1 h/session, 12 sessions for 4 weeks) + tele-VRRS (Tele@H-VRRS: 1 h/session, 36 sessions for 3 months)	[Usual care group] face-to-face VRRS: 1 h/session, 12 sessions for 4 weeks + Tele@H-unstructed cognitive stimulation: 1 h/session, 3 sessions/week for 3 months	T0: Baseline T1: 1 month T2: 4 months T3: Follow-up (7 months)	neuropsychological battery -MMSE, B.A.D.A., BADL, IADL, GDS, Everyday Memory Questionnaire -NPI [attention] TMT-A [executive function] TMT-B [working memory] FCSRT [visual spatial memory] CDT [verbal memory] RAVLT [QoL] QoL-AD	Clinic-VRRS was more efficient improving memory (FCSRT), language, attention (TMT A) and visuo-constructional abilities (CDT).	
Nousia (25)	Evaluation of the efficacy and feasibility of a telerehabilitation program using language and cognitive exercises in multi-domain amnestic mild cognitive impairment	Archives of Clinical Neuropsychology	2023	RCT	Zoom-Rehacom (telerehabilitation): 1 h/session, 2 sessions/week for 15 weeks	[Usual care group] Usual standard clinical care	Baseline (1 week before the beginning of program) 1 week after the completion of the sessions (week 15)	[general cognitive function] MoCA [attention] TMT-A [visual spatial memory] CDT [executive function] TMT-B [working memory] DSF, DSB	Training group after the telerehabilitation improved: delayed and working memory, confrontation naming, verbal fluency, and global cognition. A significant impact of the telerehabilitation program on memory (delay and working), language (naming and verbal fluency), global cognition performance.	
Rossetto (16)	A digital health home intervention for people within the Alzheimer’s disease continuum: results from the ability-telerehabilitation pilot randomized controlled trial	Annals of Medicine	2023	RCT	ABILITY condition (digital telerehabilitation platform for cognitive exercise, video tutorials for motor activities, adaptive incremental difficulty level): cognitive activities: 20 ~ 30 min/session, 5 sessions/week for 6 weeks + motor exercises: 15 ~ 25 min/session, 3 sessions/week, for 6 weeks	[Usual care group] Treatment as Usual intervention (standard manner, paper and pencil activities for cognitive exercise, written instruction for motor activities, fixed incremental difficulty level): cognitive activities: 20 ~ 30 min/session, 5 sessions/week, for 6 weeks, + motor exercises: 15 ~ 25 min/session, 3 sessions/week for 6 weeks	T0: Baseline T1: After 6 weeks of treatment T2: After 12 months after baseline	[general cognitive function] MoCA [attention] TMT-A [executive functions] TMT-B [verbal memory] CAT, DFR DTR, IFR, ITR	Treatment effect (ABILITY>Treatment as Usual): global cognitive level, especially in executive functions, and memory domains. Treatment carry-over effect (1-year follow-up): ABILITY group compared to control group, improved global cognitive functions, decreased behavioral symptoms, and caregiver distress	ABILITY program was efficient: Adherence (81% vs. 62%), higher perceived fit of demands and skills (*p* < 0.05), good level of technology usability. Data unavailable.
Pino (26)	Virtual coach and telerehabilitation for Parkinson’s disease patients: vCare system	Journal of Public Health	2024	RCT	vCare system (personalized home telerehabilitation with virtual coach system; motor and cognitive rehabilitation) : 20–45 min/session 4 sessions (2 motor sessions and 2 cognitive sessions) for 16 weeks	[Usual care group] : Standard clinical care	T0 (Pre-intervention) T1 (post-intervention)	[general cognitive function] MoCA [QoL] EQs 5D-5L (Euro Quality of Life 5 Levels) [ADLs] Schwab and England Activities of Daliy Living	Regarding intra-group differences, the vCare group showed statistically significant differences after treatment compared to pre-treatment, showing improvements in general cognitive status measured with MoCA (z = −2.4; *p* = 0.016) The usual care group showed no significant differences after intervention in any of the domains assessed	
Torpil (21)	The effectiveness of cognitive rehabilitation intervention with the telerehabilitation method for amnestic mild cognitive impairment: a feasibility randomized controlled trial	Journal of Telemedicine and Telecare	2023	RCT	Telerehabilitation (with Zoom, WhatsApp video conference, or Skype) (TR): 45 min/session, 2 sessions/week for 12 weeks	[face-to-face treatment group]: 45 min/session, 2 sessions/week for 12 weeks	Pre-intervention Post-intervention (12 weeks)	[attention, visual spatial memory] LOTCA-G scores (Orientation, Visual perception, Spatial perception, Motor praxis, Visuomotor, Thinking operation, Memory, Attention/concentration)	Cognitive skills: increased in both groups (*p* < 0.001) Within-group analysis showed a significant increase in all functions in both groups (*p* < 0.001). A statistically significant difference was observed between the groups in the post-intervention visual–spatial perception, praxis, and total LOTCA-G scores (*p* < 0.01). higher scores at face-to-face treatment group	The researchers noted that telerehabilitation is not inferior to traditional face-to-face approaches in terms of effectiveness, validity, reliability, and patient satisfaction, and even in cost, time, and accessibility for both therapists and clients
Torrisi (17)	Using telerehabilitation to improve cognitive function in post-stroke survivors: is this the time for the continuity of care?	International Journal of Rehabilitation Research	2019	RCT	Experimental group (EG) [VRRS-Evo (telerehabilitation)]: 50 min/session, 5 sessions/week for 12 weeks after discharge - VRRS Home Tablet 50 min/session, 3 sessions/week for 12 weeks	[Usual care group] (CG) Using paper–pencil tools, 50 min/session, 5 sessions/week for 12 weeks → traditional training, 50 min/session, 3 sessions/week for 12 weeks	T0: Baseline T1: After 12 weeks T2: After 24 weeks (end of protocol)	[general cognitive function] MoCA [attention] AM [attention] TMT-A [executive function] TMT-B TMT-B-A [executive function] FAB [executive function] Weigl test [working memory] Digit Span [verbal memory] RAVLT [depression] HRS-D [anxiety] HRS-A	Significant improvements were shown at the Experimental group compared to usual care group: MoCA, AM, TMT-B, TMT-B-A, RAVL.I, HRS-D, HRS-A No effects: TMT-A, RAVL.D, digit span, Weigl, FAB	
van der Linden (18)	eHealth cognitive rehabilitation for brain tumor patients: results of a randomized controlled trial	Journal of Neuro-Oncology	2021	RCT	ReMind (eHealth cognitive rehabilitation) 3 h/week for 10 weeks	[Usual care group] Waiting-list control group	T0: Before surgery T3: After 3 months T6: After 6 months T12: After 12 months	[general cognitive function] The computerized neuropsychological test battery Central Nervous System Vital Signs (CNS VS, LCC, Morrisville, North Carolina) [general cognitive function] CFQ [executive function] BRIEF-A [depression, anxiety] HADS	Proportions of participants with impairment in cognitive performance were not significantly different between the groups at T3 and T6, with percentages lying around 70% (Table 3). At T12, significantly fewer participants in the intervention group showed cognitive impairment (35% vs. 68%, *p* = 0.027). Performance-based cognitive outcome, patient-reported outcomes: not significantly differ in group means over time nor RCIs [intervention (final *n* = 20) / control group (final *n* = 25)] No significant effects were demonstrated, while adherence and satisfaction with the eHealth program were good. In clinical practice, ReMind may be helpful, if timing would be adapted to patients’ needs.	All participants found a tablet-app suitable for delivery of cognitive rehabilitation and 90% rated the program as “good” or “excellent”
Vilou (27)	Computerized cognitive rehabilitation for treatment of cognitive impairment in multiple sclerosis: an explorative study	Journal of Integrative Neuroscience	2020	RCT	BrainHQ™ (Web-based cognitive rehabilitation program): 40 min/session, 2 sessions/week for 6 weeks	Usual care	Baseline Post-treatment examinations (week 6)	[attention] TMT-A [executive function] TMT-B [executive function] Stroop word color test [verbal memory] GVLT [visual memory] BVMT-R [speed] SDMT [Depression] BDI-FS	Within-group comparisons revealed significant improvements in verbal learning (GVLT, *p* < 0.001), visuospatial memory (BVMT-R, *p* = 0.001), visual attention (TMT-A, *p* < 0.001), task switching (TMT-B, *p* < 0.001), reading speed and response inhibition (Stroop tests, *p* = 0.002) within the intervention group When group comparisons were tested by considering individual score changes across follow-up, significantly beneficial effect sizes of the intervention were noted for verbal learning (GVLT, large effect size), visuospatial memory (BVMT-R, moderate effect size), reading speed and response inhibition (Stroop tests, moderate effect size) and visual attention	
Wilson (9)	Home-based (virtual) rehabilitation improves motor and cognitive function for stroke patients: a randomized controlled trial of the Elements (EDNA-22) system	Journal of NeuroEngineering and Rehabilitation	2021	RCT	EDNA™ (tele-computerized cognitive rehabilitation program) + Treatment as Usual: 30 min/session, 3 ~ 4 sessions/week for 8 weeks	[Usual care group] GRASP (Tele-Computerized motor rehabilitation program) + Treatment as Usual: 30 min/session, 3 ~ 4 sessions/week for 8 weeks	Baseline Posttreatment examinations (week 8) Follow-up (month 3)	[general cognitive function] MoCA [Health-related QOL] SIS [Depression] NFI	For EDNA training, the pre-post effect size on the MoCA was moderate (*g* = 0.70), and triple that of the GRASP training Moderate (but non-significant) improvement in functional behavior on the SIS (*g* = 0.57) and NFI (*g* = 0.49)	

The grey shading in the table indicates the studies excluded from the meta-analysis. RCT; randomized controlled trial, VRRS; virtual reality rehabilitation system, MoCA; Montreal Cognitive Assessment, FAB; Frontal Assessment Battery, ADLs; activities of daily living, BI; Barthel Index, QOL; quality of life, SF-36; Short Form Health Survey 36, FTF; Face-to-face treatment, BDI-2; Beck Depression Inventory II, PGWBI; Psychological General Well-Being Index, CBI; Caregiver Burden Inventory, RAVLT; Rey Auditory Verbal Learning Test, EDO-10; Oblivion Detection Scale, MMQ; Multifactorial Memory Questionnaires, GDS; Geriatric Depression Scale, DASS-21; Depression, Anxiety and Stress Scale, NPI-Q; Neuropsychiatric Inventory Questionnaire, FAQ; Functional Activities Questionnaire, ACR; Adaptive cognitive remediation, DSB; Digit Span Backward, SRT; selective reminding test, BVMT-R; Brief Visuospatial Memory Test-Revised, SD; standard deviation, LSS; lexical-sematic stimulation, MMSE; Mini-Mental State Examination, DSF; Digit Span Forward, ROCF; Rey–Osterrieth Complex Figure Copy Test, RBMT-GMI; Rivermead Behavioral Memory Test-Third Edition- Global Memory Index, CFQ; Cognitive Failures Questionnaire, FrSBe; Frontal Symptoms Behavioral Scale, SF-12; Short-Form 12 Physical/Mental Component Score, TIADL; Timed Instrumental Activities of Daily Living, B.A.D.A; Battery for Analysis of Aphasic Deficits, BADL; Basic activity of daily living, IADL; Instrumental Activities of Daily Living, TMT-A; Trail Making Test A, TMT-B; Trail Making Test B, FCSRT; Free and Cued Selective Reminding Test, CDT; Clock Drawing Test, QoL-AD; Quality of Life in Alzheimer’s disease, CAT; categorical verbal fluencies, DFR; Delayed Free Recall, DTR; Delayed Total Recall, IFR; Immediate Free Recall, ITR; Immediate Total Recall, LOTCA-G; Loewenstein Occupational Therapy Cognitive Assessment–Geriatric, AM; Attentive Matrices, HRS-D; Hamilton Rating Scale-Depression, HRS-A; Hamilton Rating Scale-Anxiety, RAVL.I; Rey Auditory verbal learning. Immediate, RAVL.D; Rey Auditory verbal learning. Delay, BRIEF-A, Behavior Rating Inventory of Executive Function, HADS; Hospital Anxiety and Depression Scale, RCI; Reliable change indices, GVLT; Greek Verbal Learning Test, SDMT; Symbol Digit Modalities Test, BDI-FS; Beck depression inventory- Fast Screen, SIS; Stroke Impact Scale, NFI; Neurobehavioral Functioning Inventory, ADROL; Alzheimer’s Disease Related Quality of Life.

### Search and selection

Publications were searched in PubMed, Cochrane, EMbase, CINAHL, Web of Science (WOS), Scopus, KMbase. For comprehensive literature search, the scope of the search did not specify a start date and the end date was December 10, 2024. The review included publications in English and Korean. Detailed search terms are provided in Supplementary material 1. Two reviewers (H.J, H.H) independently conducted the study selection and data extraction. During the screening phase, when the relevance to the topics is ambiguous based on the title and abstract, a partial review of full text was conducted. Studies meeting the inclusion criteria were included in the review, while those not meeting them were excluded from the review process. A discussion or 3rd reviewer was utilized to resolve conflicts. After screening, the full text was reviewed by two reviewers and excluded studies were described with reasons for their exclusion. Based upon the PRISMA 2020 checklist, the review process was described in the flow chart and the following cases were excluded during the literature screening:

1. Studies published as abstracts only, or those for which the full text is not accessible due to reasons such as being unpublished or inaccessible in the original language (non-English/ non-Korean).
2. Studies that do not correspond to the PICO criteria.
3. Studies that do not match the predefined types of research selected for this study.

### Risk of bias (RoB) assessment

Two reviewers (S.P, D.Y.K) independently reviewed the full text for quality assessment. The risk of bias of included studies was assessed using Cochrane revised tool for Risk of Bias in randomized trials (RoB 1.0) to evaluate quality of individual studies.

### Data synthesis

Treatment effects were evaluated using Mean Difference (MD) of homogeneous outcome measures or Standardized Mean Difference (SMD) when the outcomes were measured with different scales. For assessing heterogeneity intervention and outcome measures were considered, and data from the most clinically similar trials were combined for analysis. Random-effect model is used to represent an estimate of treatment effect. Subgroups analysis was done according to diagnosis.

### Statistical analysis of evidence

We performed a meta-analysis using Reviewer Manager Software 5.4 (Cochrane Collaboration, Oxford, UK). A statistical analysis for continuous variable was conducted. Heterogeneity was estimated using I^2^, which quantifies the percentage of total variation across studies. An I^2^ value greater than 50.0% was considered indicative of substantial heterogeneity. The meta-analyses employed a random effects model with the inverse variance method for continuous outcome variables.

### Assessment of certainty of evidence

The certainty of evidence was assessed using the Grading of Recommendations, Assessment, Development, and Evaluations (GRADE) method. This method categorizes the certainty of evidence as high, moderate, low, or very low. Depending on the study design, the certainty of evidence is initially determined as ‘high’, and whether the evidence level can be lowered is determined based on specific criteria. For randomized controlled trails (RCTs), 5 factors are considered: (1) risk of bias, (2) inconsistency, (3) indirectness, (4) imprecision and (5) publication bias, and the certainty of evidence can be lowered by 1 or 2 levels. These evaluations were independently conducted by two authors and then subjected through a consensus process.

## Results

### Study selection

**Figure 1:**
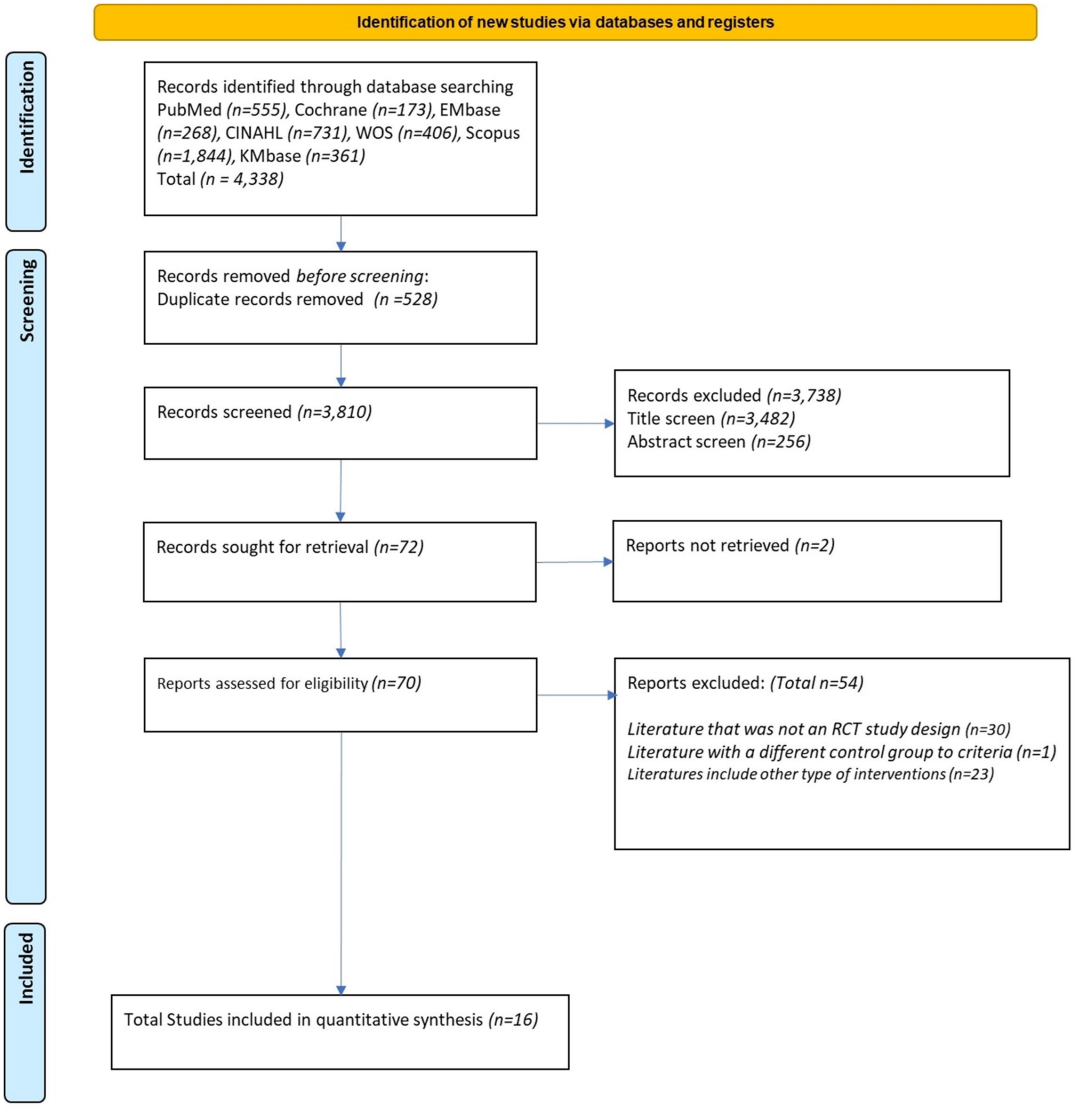
Flow diagram of the studies.

After a comprehensive literature search, 2 reviewers screened 4,338 studies for duplicate and 16 RCTs were finally selected and PRISMA flow is described in Figure 1. A description of the included studies is detailed in Table 1. Of the final selection, Charvet et al. (15), Mahncke et al. (7), Rossetto et al. (16), Torrisi et al. (17) and Van der Linden et al. (18) were excluded from the analysis because data extraction for meta-analysis was not possible. Individual data sharing requests were sent via email to the corresponding authors of these studies, but no responses were received.

### Study characteristics

**Figure 2:**
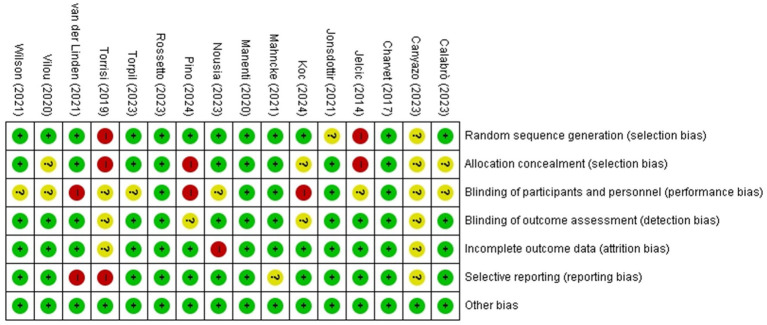
Risk of bias for included studies. The included studies were independently assessed and agreed by 2 reviewers using the Cochrane’s RoB of 1.0. The colors of the symbols represent the risk of bias as follows: green indicates a low risk of bias, yellow indicates an unclear risk of bias, and red indicates a high risk of bias.

The studies comparing the efficacy of cognitive telerehabilitation with face-to-face cognition treatment were Calabrò (19), Jelcic (20), Torpil (21). Studies comparing cognitive telerehabilitation with usual care were Canyazo (22), Jelcic (20), Jonsdottir (23), Koc (24), Manenti (12), Nousia (25), Pino (26), Vilou (27), Wilson (9). There were no available data for meta-analysis on the other outcomes, ADLs and QOL. The Risk of bias for the studies included in the analysis is shown in Figure 2.

### Meta-analysis for effects of telerehabilitation

**Figure 3:**
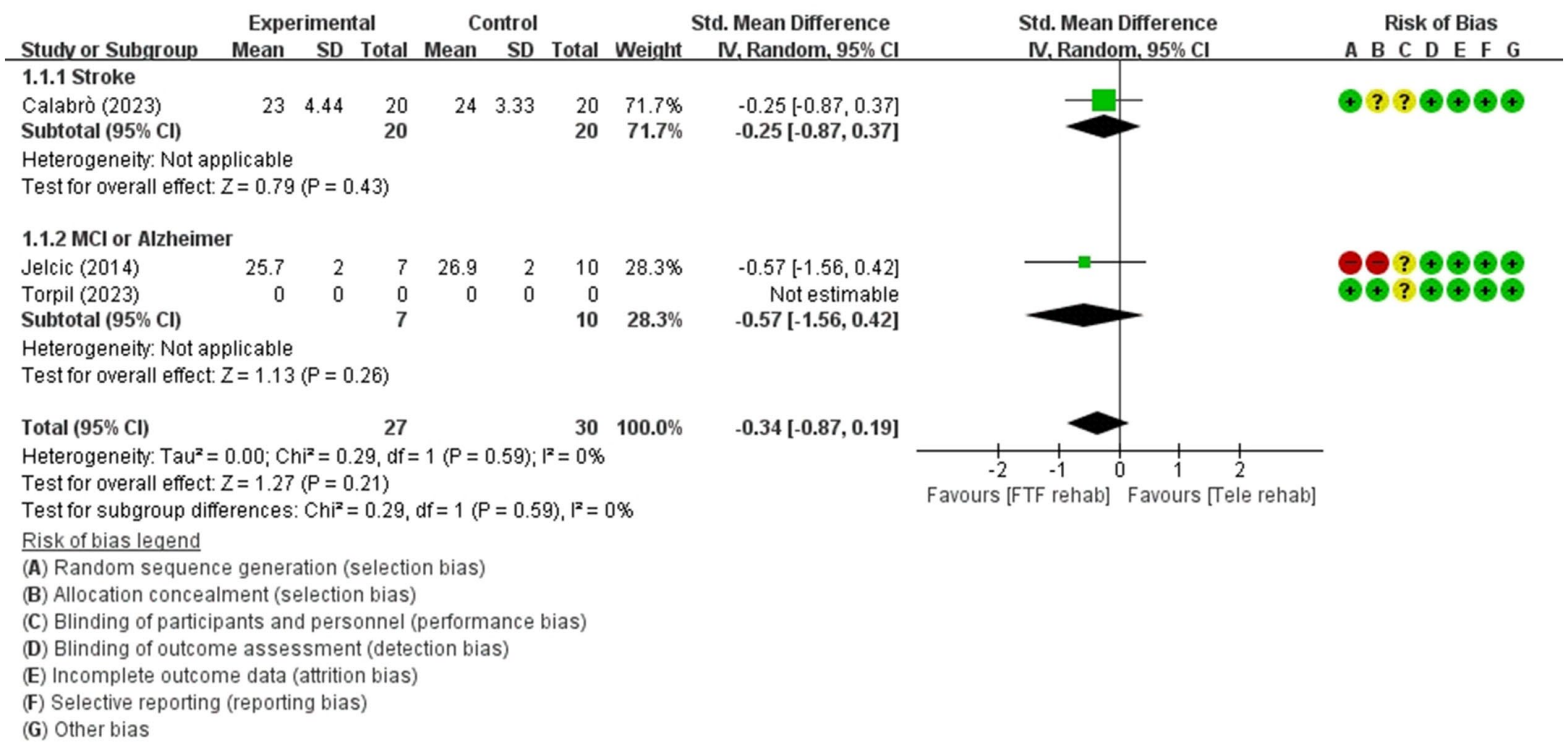
Forest plot of meta-analyses: telerehabilitation versus face-to-face treatment on global cognition (immediate). SD, standard deviation; IV, inverse-variance; CI, confidence interval.

**Figure 4:**
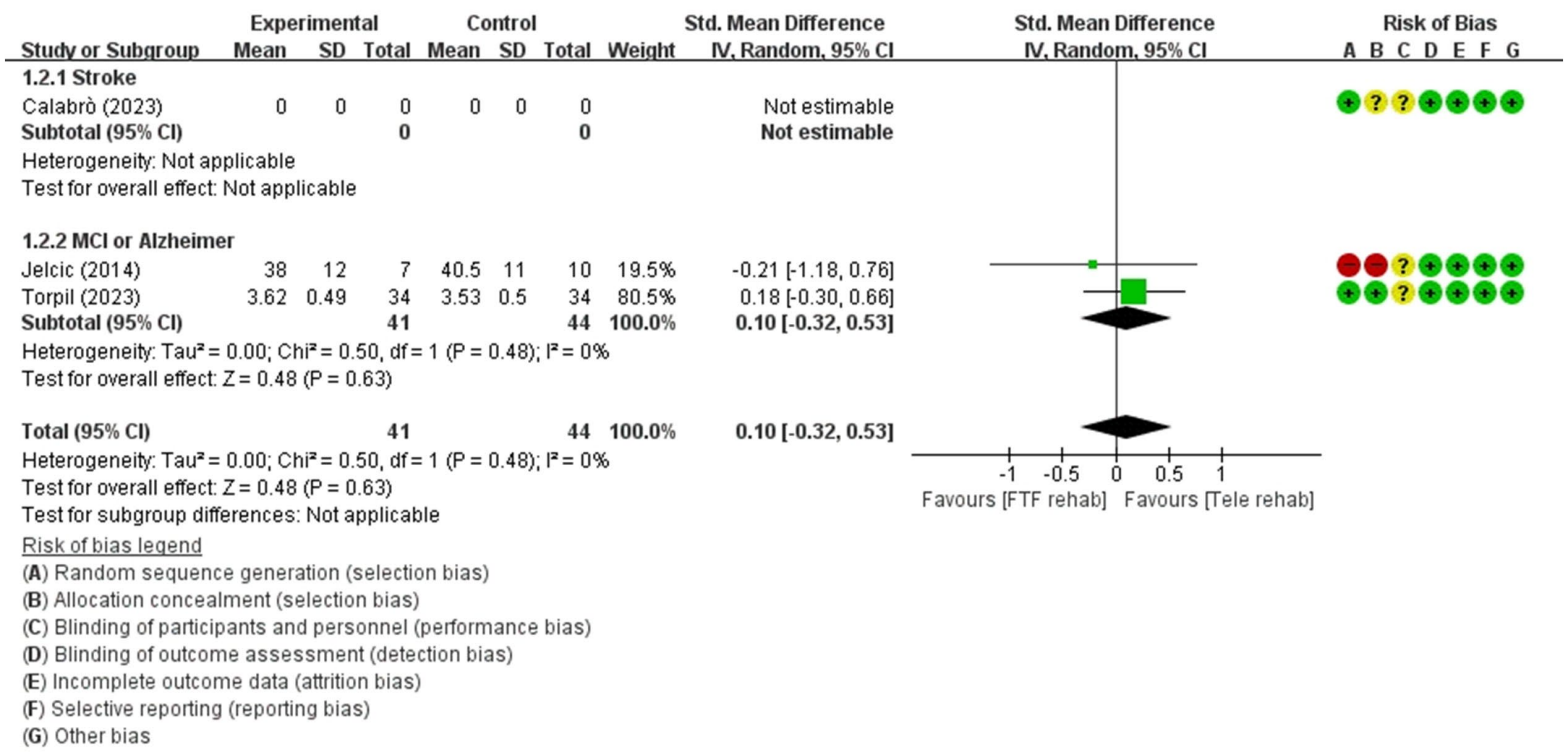
Forest plot of meta-analyses: telerehabilitation versus face-to-face treatment on attention (immediate). SD, standard deviation; IV, inverse-variance; CI, confidence interval.

**Figure 5:**
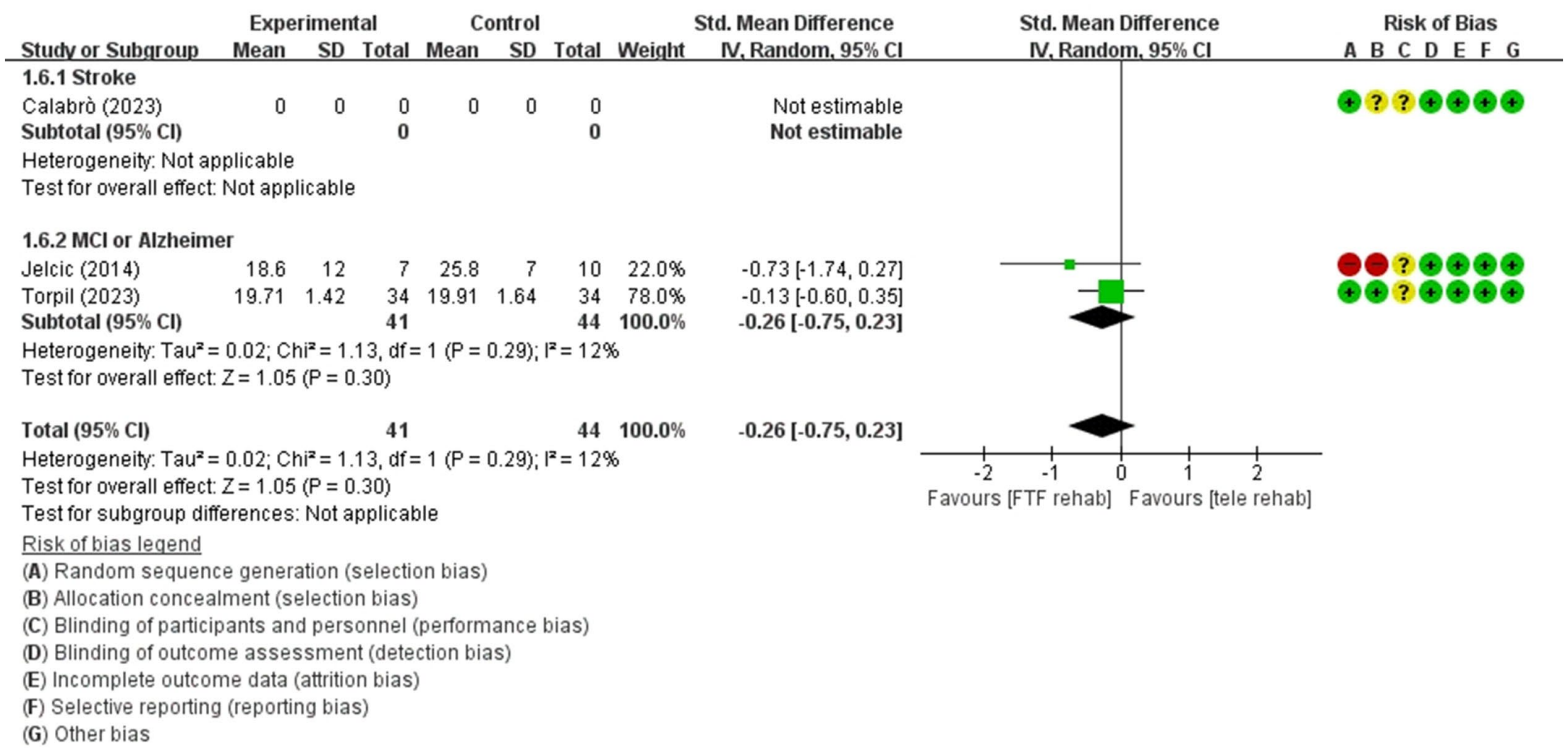
Forest plot of meta-analyses: telerehabilitation versus face-to-face treatment on visuospatial function (immediate). SD, standard deviation; IV, inverse-variance; CI, confidence interval.

**Figure 6:**
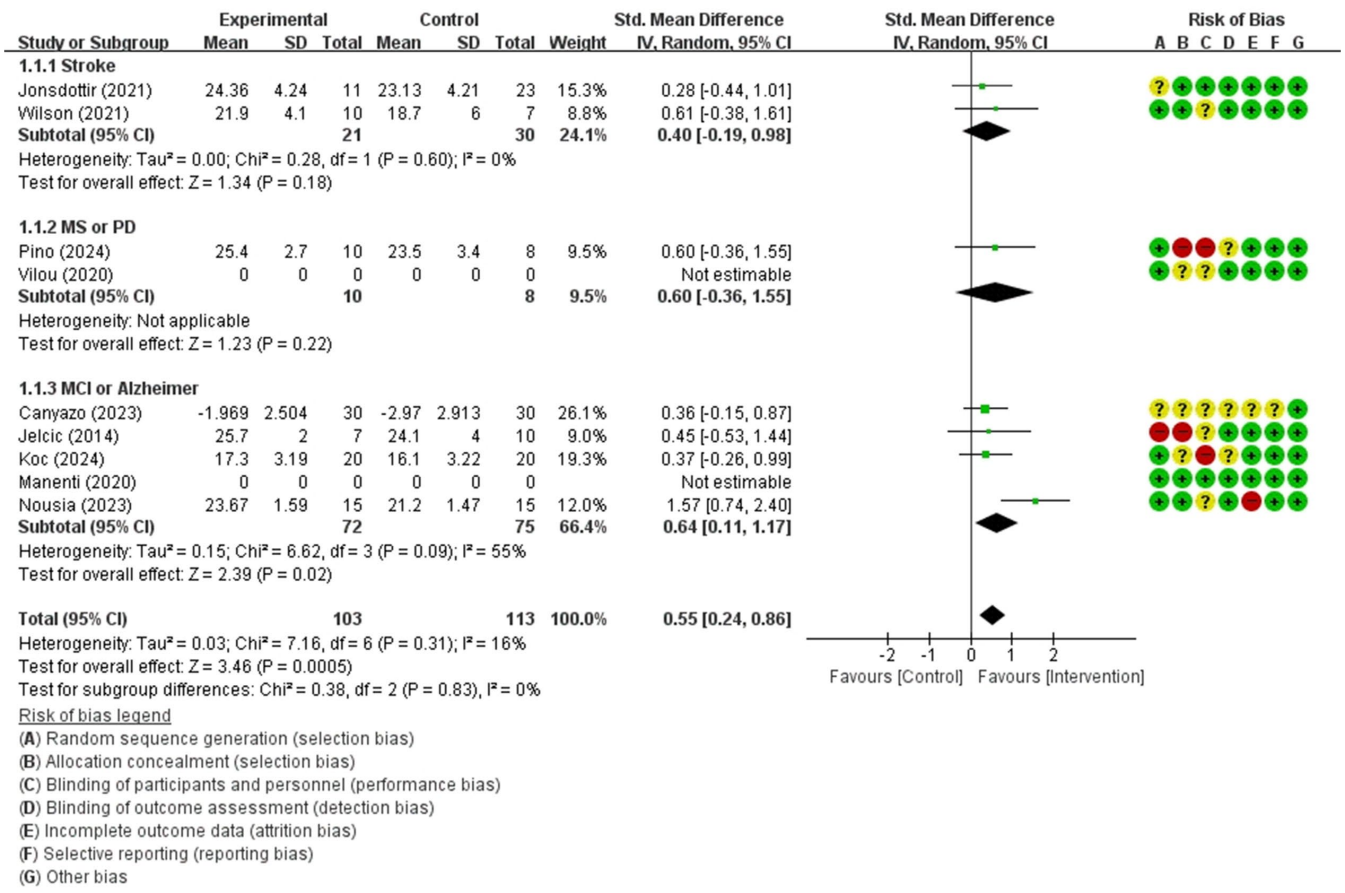
Forest plot of meta-analyses: telerehabilitation versus usual care on global cognition (immediate). SD, standard deviation; IV, inverse-variance; CI, confidence interval.

**Figure 7:**
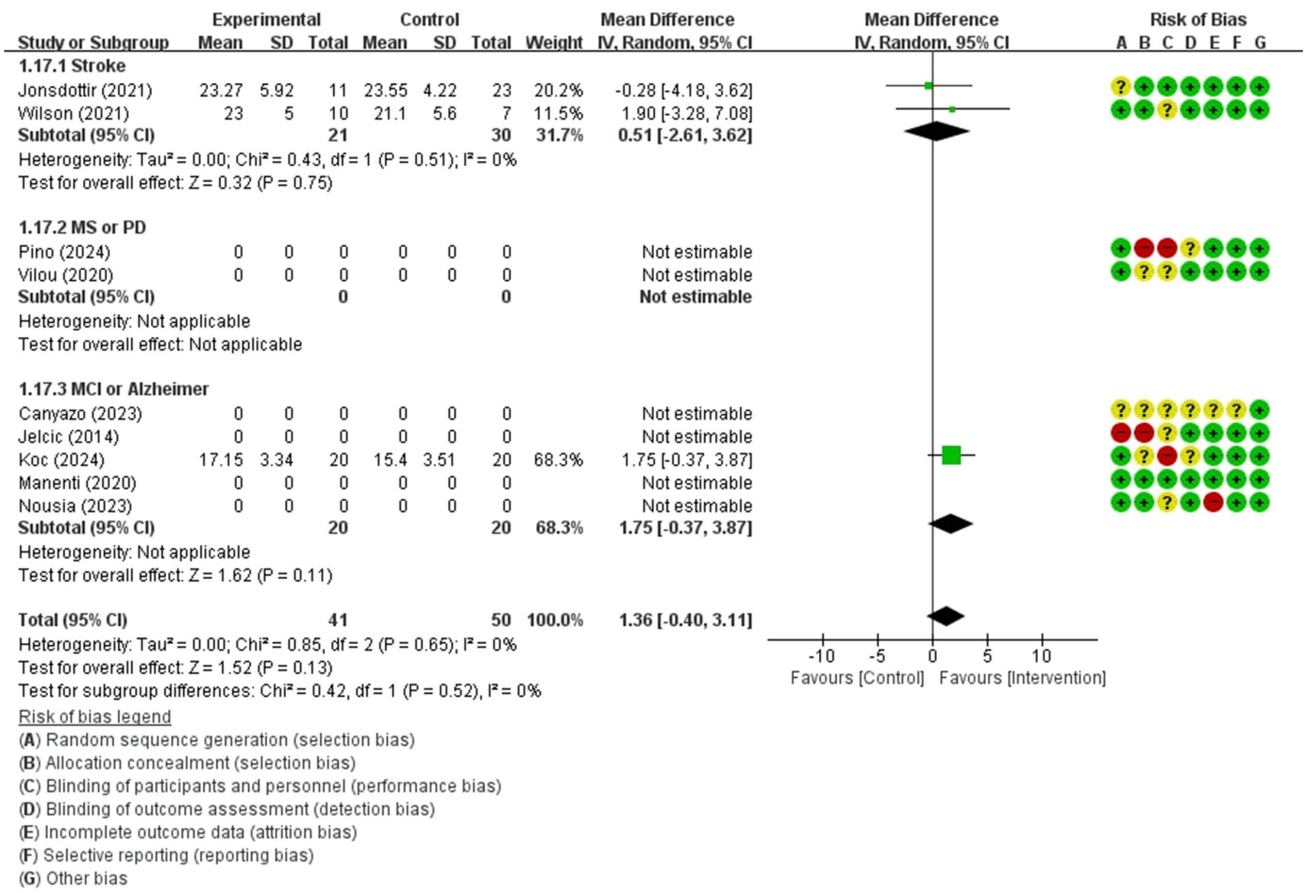
Forest plot of meta-analyses: telerehabilitation versus usual care on global cognition (persistence). SD, standard deviation; IV, inverse-variance; CI, confidence interval.

**Figure 8:**
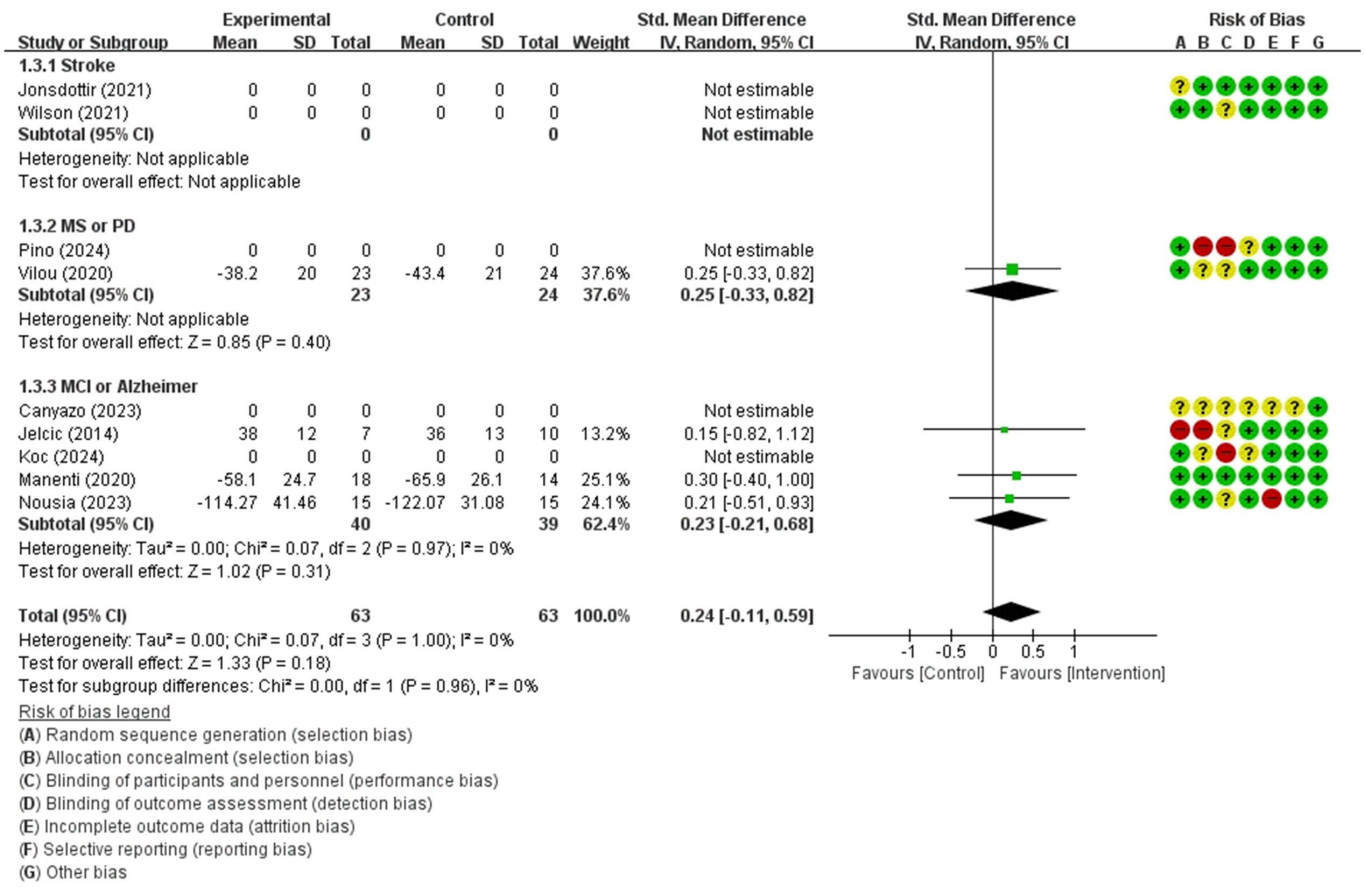
Forest plot of meta-analyses: telerehabilitation versus usual care on attention (immediate). SD, standard deviation; IV, inverse-variance; CI, confidence interval.

**Figure 9:**
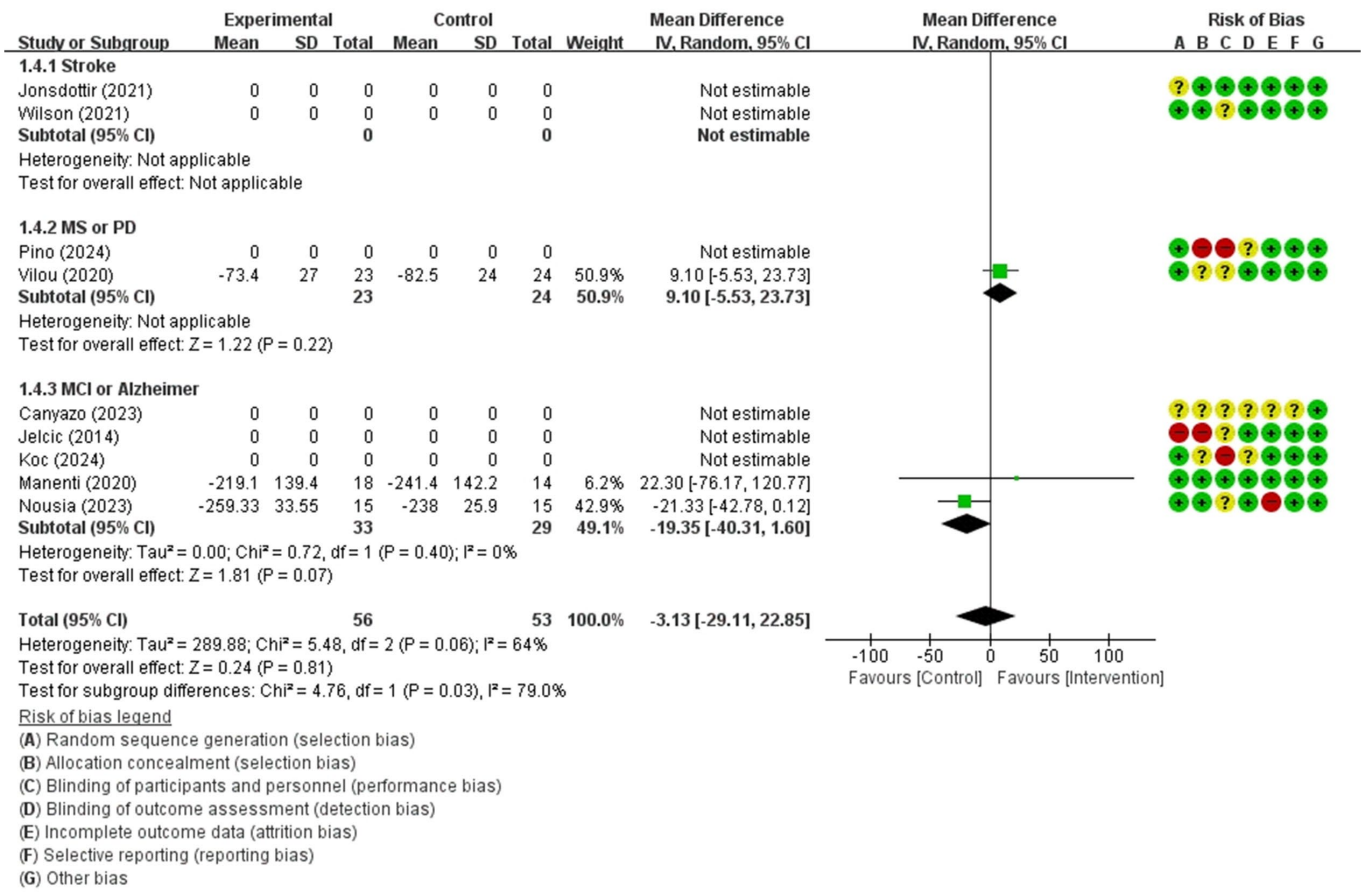
Forest plot of meta-analyses: telerehabilitation versus usual care on executive function (immediate). SD, standard deviation; IV, inverse-variance; CI, confidence interval.

**Figure 10:**
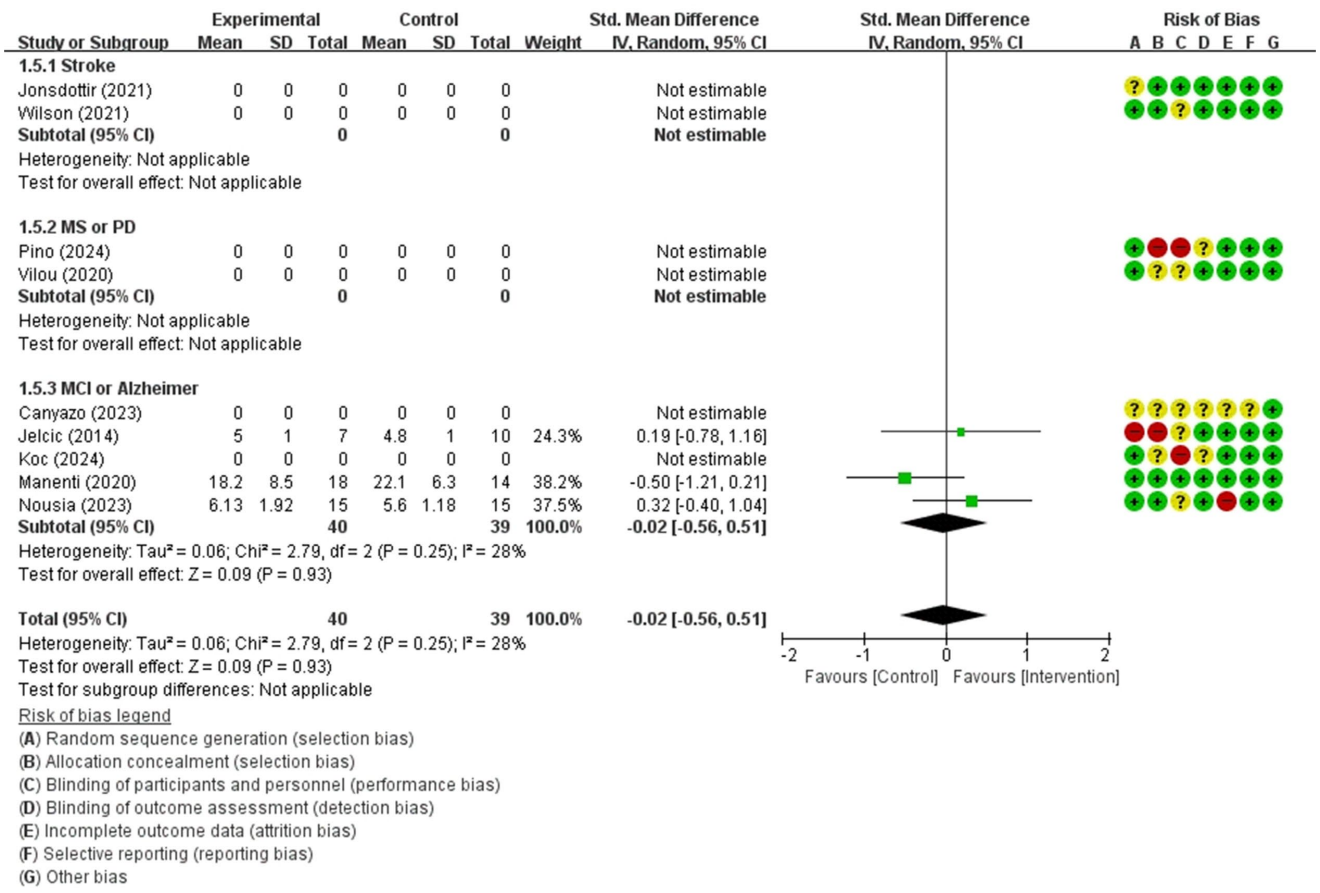
Forest plot of meta-analyses: telerehabilitation versus usual care on working memory (immediate). SD, standard deviation; IV, inverse-variance; CI, confidence interval.

**Figure 11:**
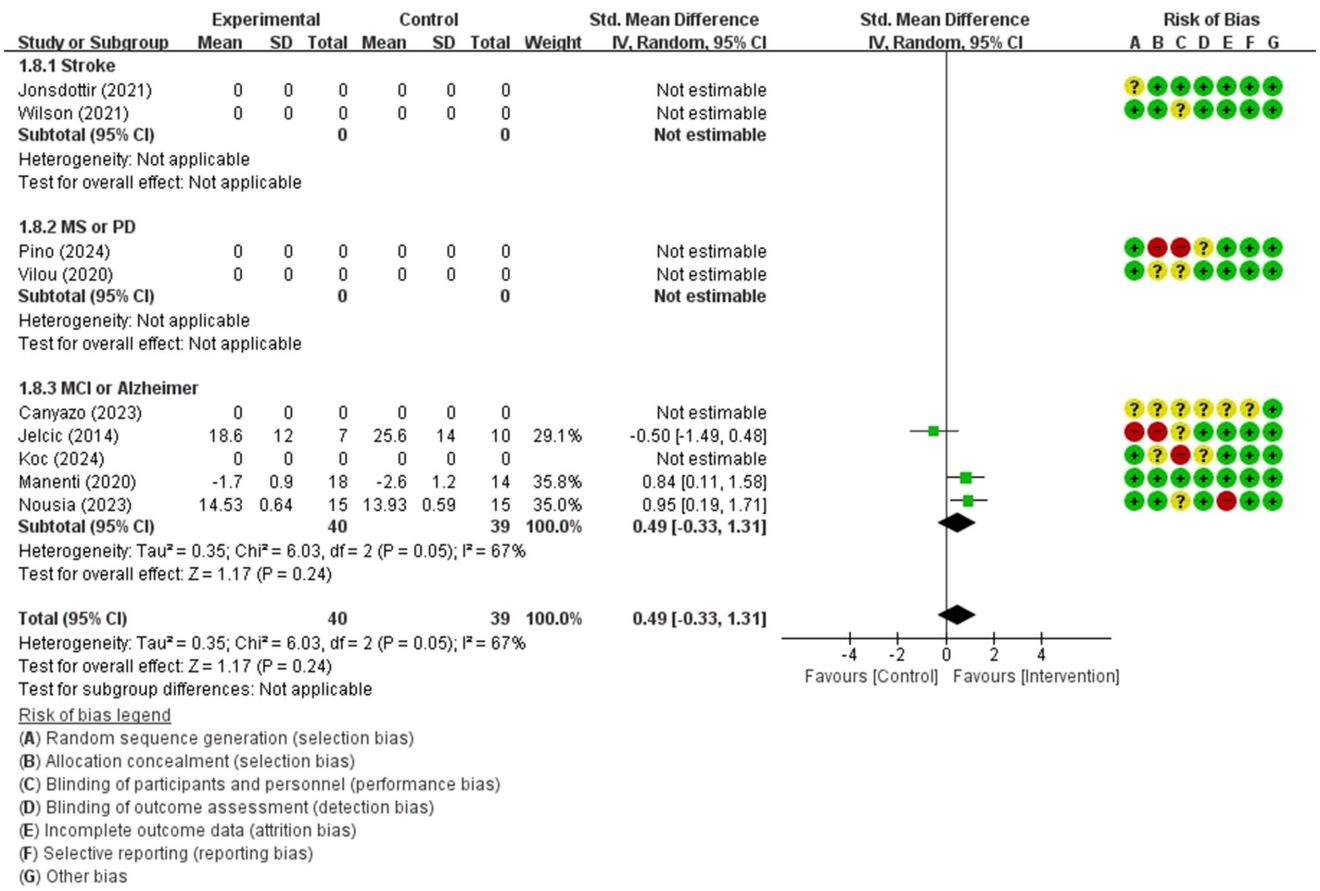
Forest plot of meta-analyses: telerehabilitation versus usual care on visuospatial function (immediate). SD, standard deviation; IV, inverse-variance; CI, confidence interval.

For comparing telerehabilitation with face-to-face cognition treatment, the meta-analysis included 2 RCTs for global cognition (immediate outcome), 2 RCTs for attention (immediate outcome), 2 RCTs for visuospatial function (immediate outcome). For comparing telerehabilitation with usual care, the meta-analysis included 7 RCTs for global cognition (immediate outcome), 3 RCTs for global cognition (persistence outcome), 4 RCTs for attention (immediate outcome), 3 RCTs for executive function (immediate outcome), 3 RCTs for working memory (immediate outcome), 3 RCTs for visuospatial function (immediate outcome). The evidence summaries and GRADE assessments are provided in Table 2, while forest plots of the meta-analyses are presented in Figures 3–11. Across all analyses, the 95% confidence intervals of the MD and SMD for the effectiveness of the cognitive telerehabilitation were distributed including zeros, indicating no significant difference between the interventions. We examined synthesis of evidence for cognitive telerehabilitation, and conducted subgroup analyses based on diagnosis, including stroke, mild cognitive impairment (MCI), Parkinsons’s disease, and multiple sclerosis. Out of the total 16 studies, 11 were included in the meta-analysis and analyzed by subdomain of cognition. The subdomain with the highest number of studies analyzed together was global cognition (immediate outcome), with 7 studies. Therefore, a funnel plot for assessing publication bias was not generated.

**Table 2:** The evidence summaries and GRADEs.

Outcomes	No. of participants / No. of studies	GRADE certainty of evidence (deduction factors)	Statistical methods (IV, Random, 95% CI)	Effect estimates
VS. face-to-face cognition treatment				
Global cognition (immediate)	57 / 2	low (Imprecision −2)	SMD	−0.34 [−0.87, 0.19]
Attention (immediate)	85 / 2	low (Imprecision −2)	SMD	0.10 [−0.32, 0.53]
Visuospatial function (immediate)	85 / 2	low (Imprecision −2)	SMD	−0.26 [−0.75, 0.23]
VS. usual care				
Global cognition (immediate)	216 / 7	moderate (Imprecision −1)	SMD	0.55 [0.24, 0.86]
Global cognition (persistence)	91 / 3	low (Imprecision −2)	MD	1.36 [−0.40, 3.11]
Attention (immediate)	126 / 4	low (Imprecision −2)	SMD	0.24 [−0.11, 0.59]
Executive function (immediate)	109 / 3	moderate (Imprecision −1)	MD	−3.13 [−29.11, 22.85]
Working memory (immediate)	79 / 3	low (Inconsistency of results −1; Imprecision −2)	SMD	−0.02 [−0.56, 0.51]
Visuospatial function (immediate)	79 / 3	very low (Inconsistency of results −1; Imprecision −2)	SMD	0.49 [−0.33, 1.31]

GRADE, Grading of Recommendations, Assessment, Development, and Evaluation; SMD, Standardized mean difference; IV, inverse-variance; CI, confidence interval; MD, mean difference.

### Telerehabilitation versus face-to-face treatment

#### Global cognition (immediate)

The studies included in the meta-analysis to determine the effects of telerehabilitation versus face-to-face treatment on global cognition (immediate), were a total of 2 studies. The evaluation tools for the outcome measures were Montreal Cognitive Assessment (MoCA) and Mini-Mental State Examination (MMSE). The effect size was calculated using SMD and the result was −0.34 (−0.87, 0.19).

In the stroke subgroup analyses, to evaluate the effects of telerehabilitation versus face-to-face treatment on global cognition (immediate), a total of 1 study was included in the meta-analysis. The evaluation tool for the outcome measures MoCA. The effect size was calculated using SMD and the result was −0.25 (−0.87, 0.37).

In the MCI or Alzheimer’s disease subgroup analyses, to evaluate the effects of telerehabilitation versus face-to-face treatment on global cognition (immediate), a total of 1 study was included in the meta-analysis. The evaluation tool for the outcome measures was MMSE. The effect size was calculated using SMD and the result was −0.57 (−1.56, 0.42) (Figure 3).

#### Attention (immediate)

For attention (immediate), a total of 2 studies were included in the meta-analysis to determine the effects of telerehabilitation versus face-to-face treatment in patients with MCI or Alzheimer’s disease. The evaluation tools were digital cancelation and Loewenstein Occupational Therapy Cognitive Assessment-Geriatric (LOTCA-G). The effect size was calculated using SMD and the result was 0.10 (−0.32, 0.53) (Figure 4).

#### Visuospatial function (immediate)

For visuospatial function (immediate), a total of 2 studies were included in the meta-analysis to determine the effects of telerehabilitation versus face-to-face treatment. The evaluation tools were Rey-Osterrieth complex figure copy test (ROCF) and LOTCA-G. The effect size was calculated using SMD and the results were − 0.26 (−0.75, 0.23) (Figure 5).

### Telerehabilitation vs usual care group

#### Global cognition (immediate)

The studies included in the meta-analysis to determine the effects of telerehabilitation versus usual care on global cognition (immediate), were a total of 7 studies. The evaluation tools of the outcome measures were MoCA and MMSE. The effect size was calculated using SMD and the result was 0.55 (0.24, 0.86).

In the stroke subgroup analyses, to evaluate the effects of telerehabilitation versus usual care on global cognition (immediate), a total of 2 studies were included in the meta-analysis. The evaluation tool for the outcome measures was MoCA. The effect size was calculated using SMD and the result was 0.40 (−0.19, 0.98).

In the multiple sclerosis or Parkinson’s disease subgroup analyses, to evaluate the effects of telerehabilitation versus usual care on global cognition (immediate), a total of 1 study was included in the meta-analysis. The evaluation tool for the outcome measures was MoCA. The effect size was calculated using SMD and the result was 0.60 (−0.36, 1.55).

In the MCI or Alzheimer’s disease subgroup analyses, to evaluate the effects of telerehabilitation versus usual care on global cognition (immediate), a total of 4 studies were included in the meta-analysis. The evaluation tools were MoCA and MMSE. The effect size was calculated using SMD and the result was 0.64 (0.11, 1.17) (Figure 6).

#### Global cognition (persistence)

The studies included the meta-analysis to determine the effects of telerehabilitation versus usual care on global cognition (persistence), were a total of 3 studies. The evaluation tool was MoCA. The effect size was calculated using MD and the result was 1.36 (−0.40, 3.11).

In the stroke subgroup analyses, to evaluate the effects of telerehabilitation versus usual care on global cognition (persistence), a total of 2 studies were included in the meta-analysis. The evaluation tool for the outcome measures was MoCA. The effect size was calculated using MD and the result was 0.51 (−2.61, 3.62).

In the MCI or Alzheimer’s disease subgroup analyses, to evaluate the effects of telerehabilitation versus usual care on global cognition (persistence), a total of 1 study was included in the meta-analysis. The evaluation tool for the outcome measures was MoCA. The effect size was calculated using MD and the result was 1.75 (−0.37, 3.87) (Figure 7).

#### Attention (immediate)

The studies included in the meta-analysis to determine the effects of telerehabilitation versus usual care on attention (immediate), were a total of 4 studies. The evaluation tools for outcome measures were Trail Making Test-A (TMT-A) and digit cancelation. The effect size was calculated using SMD and the result was 0.24 (−0.11, 0.59).

In the multiple sclerosis subgroup analyses, to evaluate the effects of telerehabilitation versus usual care on attention (immediate), a total of 1 study was included in the meta-analysis. The evaluation tool for the outcome measures was TMT-A. The effect size was calculated using SMD and the result was 0.25 (−0.33, 0.82).

In the MCI or Alzheimer’s disease subgroup analyses, to evaluate the effects of telerehabilitation versus usual care on attention (immediate), a total of 3 studies were included in meta-analysis. The evaluation tools were TMT-A and digit cancelation. The effect size was calculated using SMD and the result was 0.23 (−0.21, 0.68) (Figure 8).

#### Executive function (immediate)

The studies included in the meta-analysis to determine the effects of telerehabilitation versus usual care on executive function (immediate), were a total of 3 studies. The evaluation tool was Trail Making Test-B (TMT-B). The effect size was calculated using MD and the result was −3.13 (−29.11, 22.85).

In the multiple sclerosis subgroup analyses, to evaluate the effects of telerehabilitation versus usual care on executive function (immediate), a total of 1 study was included in meta-analysis. The evaluation tool was TMT-B. The effect size was calculated using MD and the result was 9.10 (−5.53, 23.73).

In the MCI or Alzheimer’s disease group analyses, to evaluate the effects of telerehabilitation versus usual care on executive function (immediate), a total of 2 studies were included in meta-analysis. The evaluation tool was TMT-B. The effect size was calculated using MD and the result was −19.35 (−40.31, 1.60) (Figure 9).

#### Working memory (immediate)

For working memory (immediate), a total of 3 studies were included in meta-analysis to determine the effects of telerehabilitation versus usual care in patients with MCI or Alzheimer’s disease. The evaluation tools were Free and Cued Selective Reminding test (FCSRT), digital span (Forward). The effect size was calculated using SMD and the result was −0.02 (−0.56, 0.51) (Figure 10).

#### Visuospatial function (immediate)

For visuospatial function (immediate), a total of 3 studies were included in meta-analysis to determine the effects of telerehabilitation versus usual care in patients with multiple sclerosis. The evaluation tools were Clock drawing test and ROCF. The effect size was calculated using SMD and the result was 0.49 (−0.33, 1.31) (Figure 11).

## Discussion

To determine the effectiveness of telerehabilitation, two approaches of meta-analysis were conducted. In this analysis, we categorized and compared different clinical conditions as follows: usual care was defined as no treatment or sham treatment, while face-to-face treatment referred to traditional therapy directly provided by a therapist. Telerehabilitation was defined as treatment delivered using remote devices capable of providing medical rehabilitation services. The first analysis compared cognitive telerehabilitation with traditional face-to-face cognition treatment. The outcomes showed that cognitive telerehabilitation was not significantly inferior to traditional face-to-face treatment in global cognition, attention, and visuospatial function. The second analysis compared cognitive telerehabilitation with the usual care group. Cognitive telerehabilitation demonstrated better outcomes in immediate global cognition compared to usual care. However, no significant differences were observed in persistent global cognition, attention, executive function, working memory, or visuospatial function. The studies analyzing executive function and visuospatial function showed high heterogeneity, with I^2^ values exceeding 50%. Overall, the meta-analysis results suggest that cognitive telerehabilitation offers significant benefits in improving immediate global cognition in patients with cognitive dysfunction compared to usual care or sham treatment. Additionally, it demonstrates an equivalent level of effectiveness in cognitive function improvement when compared to traditional face-to-face cognition treatment. These results could provide support for the implementation of cognitive telerehabilitation.

The results of the studies that were not included in the meta-analysis because their data could not be used were similar to the findings of the meta-analysis. In a study by Charvet et al. (15), significant improvement in cognitive function was observed in patients with multiple sclerosis when comparing a remotely monitored, supervised-based telerehabilitation group with a computer-based treatment (usual care group). They suggested that cognitive telerehabilitation could serve as an alternative method for cognitive rehabilitation through remote supervision. Mahncke et al. (7) conducted a randomized controlled trial comparing the effects of telerehabilitation versus computer game-based treatment (usual care group) on cognitive function in traumatic brain injury patients. They observed improvements in cognitive function in the telerehabilitation group, as well as improvements in depressive and cognitive symptoms in both groups. However, no significant differences were observed in instrumental activities of daily living between the two groups. Rossetto et al. (16) conducted a randomized controlled trial comparing cognitive telerehabilitation with usual care in patients with mild cognitive impairment and Alzheimer’s disease, and reported significant improvement on global cognitive level, including language, memory domains, and executive functions. It is known that impairments of executive function can have the most devastating impact on activities of daily living because of its super ordinate role in behavioral and cognitive processing (28). In addition, both patients and caregivers responded positively to the system usability scale and caregivers also noted reduced levels of distress associated with caregiving (16). Caregiver burden, defined as a multidimensional response linked to caregiver distress, can be exacerbated by various factors (29). A predictive risk factor that increases caregiver burden are known to include the overall negative experience with formal care and services (29). The psychological well-being of caregivers was linked to the nature of caregiving tasks, their subjective perception of rehabilitation, and the functional recovery of patients (30). A more positive approach by caregivers to rehabilitation was also corrected with an overall beneficial influence on the caregiving process in rehabilitation and improved functional outcomes for patients (30). Torrisi et al. (17) conducted a randomized controlled trial comparing telerehabilitation using virtual reality with usual care (usual care group) in patients with post stroke cognitive dysfunction to assess the efficacy of improving cognitive function. Significant improvements were observed in global cognitive level, attention, memory, and linguistic skills domains. The study also reported that participants perceived consistent attention and maintained a high level of motivation. Furthermore, the study emphasized the positive effects of telerehabilitation, highlighting the importance of longer training sessions facilitated by participant encouragement. Van der Linder et al. (18) conducted a randomized controlled trial comparing telerehabilitation with tablet-based cognitive rehabilitation (usual care group) in patients with brain tumors to assess cognitive function outcomes. While the outcomes did not significantly differ in group, 90% of participants reported positive feedback about the intervention, with 95% indicating that they would recommend the program to others.

The included studies observed limitations of cognitive telerehabilitation. Limitations included difficulties in using telerehabilitation devices, such as reduced user engagement in digital literacy and lack of familiarity with the device. Yi et al. (31) conducted a systematic review on the barriers and facilitators of telerehabilitation for patients with dementia. Barriers included meeting technological requirements and adapting to sensory needs. Technological barriers encompassed the lack of necessary equipment and the older adults’ ability to independently operate technologies. Sensory challenges, such as communication difficulties related to hearing and vision, were highlighted across multiple studies as barriers to the successful adoption of telemedicine. To address these barriers, enabling factors such as assistance of caregivers, pre-training on devices, utilization of captioned services to enhance communication, and the incorporation of electronic magnification and text-to-speech technology on devices were proposed.

Within the included studies, no significant adverse effects related to telerehabilitation were observed. Meanwhile, Gideon A Caplan et al. (32) conducted a randomized controlled trial comparing the incidence of delirium in in-hospital rehabilitation with early discharge rehabilitation in 104 elderly individuals and found that the incidence of delirium was lower during the rehabilitation at home process.

The effectiveness of cognitive telerehabilitation, as discussed above, shows better outcomes compared to usual care and comparable effects to face-to-face treatment. Usual care may result in less effective outcomes compared to active cognitive treatment, whereas face-to-face treatment presents spatial and temporal constraints as well as cost issues. Although telerehabilitation may pose challenges related to digital literacy in device usage, its adoption is supported by advantages such as improved accessibility and continuity of rehabilitation. Especially, it can be used while reducing time and space constraints in the individual’s own environment, and these advantages are also beneficial in preventing delirium, which can occur in elderly individuals and patients with acquired brain injuries associated with cognitive dysfunction. Telerehabilitation is not meant to replace the traditional face-to-face treatment but can be applied in a variety of ways depending on patient needs and characteristics. Cognitive telerehabilitation could prove beneficial in addressing chronic diseases with significant social implications and issues related to continuous long-term care, encompassing conditions associated with aging, such as dementia and other neurogenerative disorders. From this perspective and the results of this meta-analysis demonstrating that cognitive telerehabilitation is not inferior to face-to-face treatment and is more effective than usual care in improving general cognition (immediate), it may, in certain circumstances, even be superior to face-to-face treatment, particularly in terms of cost and accessibility.

## Data Availability

The original contributions presented in the study are included in the article/Supplementary material, further inquiries can be directed to the corresponding author.

## Footnotes

^1^ https://www.crd.york.ac.uk/prospero/display_record.php?ID=CRD42023454250
