# Supplementary material for: A systematic review of cognitive telerehabilitation in patients with cognitive dysfunction
Source: Front Neurol. 2025 Jan 15;15:1450977. doi: 10.3389/fneur.2024.1450977 (PMC11774910; doi:10.3389/fneur.2024.1450977)
Supplement: Supplementary file 1 [file Table_1.docx]

**Detailed search strategies for online databases**

- Search strategy of Pubmed
  - (stroke OR “traumatic brain injury“ OR “brain injuries, traumatic“ OR “brain lesion, traumatic“ OR “brain system trauma“ OR “brain trauma” OR “cerebral trauma” OR “cerebrovascular trauma” OR “encephalopathy, traumatic” OR “mild traumatic brain injury” OR “organic cerebral trauma” OR “posttraumatic encephalopathy” OR “traumatic brain injuries” OR “traumatic brain injury” OR “traumatic brain lesion” OR “traumatic cerebral lesion” OR “traumatic encephalopathy” OR “degenerative disease” OR “degenerative disease” OR “neurodegenerative disease” OR “neurodegenerative diseases” OR “cognitive defect” OR “cognition disorder” OR “cognition disorders” OR “cognitive defect” OR “cognitive defects” OR “cognitive deficit” OR “cognitive disability” OR “cognitive disorder” OR “cognitive disorders” OR “cognitive dysfunction” OR “cognitive impairment” OR “delirium, dementia, amnestic, cognitive disorders” OR “overinclusion” OR “response interference”) AND (telerehabilitation OR “e-rehabilitation” OR “remote rehabilitation” OR “tele-rehabilitation” OR “telerehabilitation” OR “virtual rehabilitation”) AND (cognition OR “cognition” OR “cognitive accessibility” OR “cognitive balance” OR “cognitive dissonance” OR “cognitive function” OR “cognitive structure” OR “cognitive symptoms” OR “cognitive task” OR “cognitive thinking” OR “neurobehavioural manifestations” OR “volition” OR “attention” OR “attention” OR “attentiveness” OR “memory” OR “item recall” OR “memory” OR “memory function” OR “nonspatial memory” OR “remembering” OR “reminiscence” OR “executive function” OR “cognitive control” OR “executive control” OR “executive function” OR “depth perception” OR “depth discrimination” OR “depth perception” OR “perception, depth” OR “perception, space” OR “perception, visuospatial” OR “space perception” OR “spatial perception” OR “visio-spatial perception” OR “vision disparity” OR “visiospatial perception” OR “visuo-spatial perception” OR “visuospatial perception” OR “spatial attention” OR “spatial attention” OR “spatial visual attention” OR “visiospatial attention” OR “visual spatial attention” OR “visuo-spatial attention” OR “visuospatial attention” OR “visuospatial function” OR “visuospatial neglect” OR “daily life activity” OR “adl (activities of daily living)” OR “activities of daily living” OR “activity, daily living” OR “daily life activity” OR “daily living activity” OR “european quality of life 5 dimensions questionnaire” OR “short form 36” OR “36 item short form health survey” OR “sf-36” OR “sf36” OR “short form 36” OR “short form 36 health survey” OR “quality of life” OR “hrql” OR “health related quality of life” OR “life quality” OR “quality of life”)
- Search strategy of Embase
  - (stroke OR 'traumatic brain injury'/exp OR 'brain injuries, traumatic' OR 'brain lesion, traumatic' OR 'brain system trauma' OR 'brain trauma' OR 'cerebral trauma' OR 'cerebrovascular trauma' OR 'encephalopathy, traumatic' OR 'mild traumatic brain injury' OR 'organic cerebral trauma' OR 'posttraumatic encephalopathy' OR 'traumatic brain injuries' OR 'traumatic brain injury' OR 'traumatic brain lesion' OR 'traumatic cerebral lesion' OR 'traumatic encephalopathy' OR 'degenerative disease'/exp OR 'degenerative disease' OR 'neurodegenerative disease' OR 'neurodegenerative diseases' OR 'cognitive defect'/exp OR 'cognition disorder' OR 'cognition disorders' OR 'cognitive defect' OR 'cognitive defects' OR 'cognitive deficit' OR 'cognitive disability' OR 'cognitive disorder' OR 'cognitive disorders' OR 'cognitive dysfunction' OR 'cognitive impairment' OR 'delirium, dementia, amnestic, cognitive disorders' OR 'overinclusion' OR 'response interference') AND ('telerehabilitation'/exp OR 'e-rehabilitation' OR 'remote rehabilitation' OR 'tele-rehabilitation' OR 'telerehabilitation' OR 'virtual rehabilitation') AND ('cognition'/exp OR 'cognition' OR 'cognitive accessibility' OR 'cognitive balance' OR 'cognitive dissonance' OR 'cognitive function' OR 'cognitive structure' OR 'cognitive symptoms' OR 'cognitive task' OR 'cognitive thinking' OR 'neurobehavioural manifestations' OR 'volition' OR 'attention'/exp OR 'attention' OR 'attentiveness' OR 'memory'/exp OR 'item recall' OR 'memory' OR 'memory function' OR 'nonspatial memory' OR 'remembering' OR 'reminiscence' OR 'executive function'/exp OR 'cognitive control' OR 'executive control' OR 'executive function' OR 'depth perception'/exp OR 'depth discrimination' OR 'depth perception' OR 'perception, depth' OR 'perception, space' OR 'perception, visuospatial' OR 'space perception' OR 'spatial perception' OR 'visio-spatial perception' OR 'vision disparity' OR 'visiospatial perception' OR 'visuo-spatial perception' OR 'visuospatial perception' OR 'spatial attention'/exp OR 'spatial attention' OR 'spatial visual attention' OR 'visiospatial attention' OR 'visual spatial attention' OR 'visuo-spatial attention' OR 'visuospatial attention' OR 'visuospatial function'/exp OR 'visuospatial neglect'/exp OR 'daily life activity'/exp OR 'adl (activities of daily living)' OR 'activities of daily living' OR 'activity, daily living' OR 'daily life activity' OR 'daily living activity' OR 'european quality of life 5 dimensions questionnaire'/exp OR 'short form 36'/exp OR '36 item short form health survey' OR 'sf-36' OR 'sf36' OR 'short form 36' OR 'short form 36 health survey' OR 'quality of life'/exp OR 'hrql' OR 'health related quality of life' OR 'life quality' OR 'quality of life') AND ('randomized controlled trial'/exp OR 'controlled clinical trial (topic)'/exp OR 'controlled clinical trial (topic)' OR 'controlled clinical trials' OR 'controlled clinical trials as topic' OR 'non randomized controlled trials as topic' OR 'non-randomized controlled trials as topic' OR 'grey literature'/exp) NOT 'case report'/exp NOT ('case study'/exp OR 'case series' OR 'case studies' OR 'case study' OR 'large case series')
- Search strategy of Cochrane

#1 MeSH descriptor: [Stroke] explode all trees

#2 stroke

#3 MeSH descriptor: [Traumatic brain injury] explode all trees

#4 traumatic brain injury

#5 MeSH descriptor: [Neurodegenerative diseases] explode all trees

#6 neurodegenerative diseases

#7 MeSH descriptor: [Cognitive Dysfunctions] explode all trees

#8 cognitive dysfunctions

#9 {OR #1-#8}

#10 MeSH descriptor: [Telemedicine] explode all trees

#11 telemedicine

#12 MeSH descriptor: [Telerehabilitation] explode all trees

#13 telerehabilitation

#14 {OR #10-#13}

#15 MeSH descriptor: [Cognition] explode all trees

#16 cognition

#17 MeSH descriptor: [Memory] explode all trees

#18 memory

#19 MeSH descriptor: [Attention] explode all trees

#20 attention

#21 MeSH descriptor: [Executive Function] explode all trees

#22 executive function

#23 MeSH descriptor: [Quality of Life] explode all trees

#24 Quality of Life

#25 MeSH descriptor: [Activities of Daily Living] explode all trees

#26 Activities of Daily Living

#27 {OR #15-#26}

#28 #9 AND #14 AND #27

- Search strategy of CINAHL
  - (stroke OR “traumatic brain injury“ OR “brain injuries, traumatic“ OR “brain lesion, traumatic“ OR “brain system trauma“ OR “brain trauma” OR “cerebral trauma” OR “cerebrovascular trauma” OR “encephalopathy, traumatic” OR “mild traumatic brain injury” OR “organic cerebral trauma” OR “posttraumatic encephalopathy” OR “traumatic brain injuries” OR “traumatic brain injury” OR “traumatic brain lesion” OR “traumatic cerebral lesion” OR “traumatic encephalopathy” OR “degenerative disease” OR “degenerative disease” OR “neurodegenerative disease” OR “neurodegenerative diseases” OR “cognitive defect” OR “cognition disorder” OR “cognition disorders” OR “cognitive defect” OR “cognitive defects” OR “cognitive deficit” OR “cognitive disability” OR “cognitive disorder” OR “cognitive disorders” OR “cognitive dysfunction” OR “cognitive impairment” OR “delirium, dementia, amnestic, cognitive disorders” OR “overinclusion” OR “response interference”) AND (telerehabilitation OR “e-rehabilitation” OR “remote rehabilitation” OR “tele-rehabilitation” OR “telerehabilitation” OR “virtual rehabilitation”) AND (cognition OR “cognition” OR “cognitive accessibility” OR “cognitive balance” OR “cognitive dissonance” OR “cognitive function” OR “cognitive structure” OR “cognitive symptoms” OR “cognitive task” OR “cognitive thinking” OR “neurobehavioural manifestations” OR “volition” OR “attention” OR “attention” OR “attentiveness” OR “memory” OR “item recall” OR “memory” OR “memory function” OR “nonspatial memory” OR “remembering” OR “reminiscence” OR “executive function” OR “cognitive control” OR “executive control” OR “executive function” OR “depth perception” OR “depth discrimination” OR “depth perception” OR “perception, depth” OR “perception, space” OR “perception, visuospatial” OR “space perception” OR “spatial perception” OR “visio-spatial perception” OR “vision disparity” OR “visiospatial perception” OR “visuo-spatial perception” OR “visuospatial perception” OR “spatial attention” OR “spatial attention” OR “spatial visual attention” OR “visiospatial attention” OR “visual spatial attention” OR “visuo-spatial attention” OR “visuospatial attention” OR “visuospatial function” OR “visuospatial neglect” OR “daily life activity” OR “adl (activities of daily living)” OR “activities of daily living” OR “activity, daily living” OR “daily life activity” OR “daily living activity” OR “european quality of life 5 dimensions questionnaire” OR “short form 36” OR “36 item short form health survey” OR “sf-36” OR “sf36” OR “short form 36” OR “short form 36 health survey” OR “quality of life” OR “hrql” OR “health related quality of life” OR “life quality” OR “quality of life”)
- Search strategy of SCOPUS
  - (stroke OR "Traumatic brain injury" OR "TBI" OR "neurodegenerative diseases" OR "cognitive dysfunction" ) AND ( telerehabilitation ) AND ( cognition OR "Memory" OR "attention" OR "Executive function" OR "visuospatial perception" OR "activities of daily livings" OR "ADL" OR "Quality of life" OR "QOL" OR "european quality of life 5 dimensions questionnaire" OR " EQ-5D" OR "short form 36" OR "sf-36" OR "hrql" OR "health related quality of life" ) AND ( LIMIT-TO ( LANGUAGE , "English" ) ) AND ( EXCLUDE ( EXACTKEYWORD , "Systematic Review" ) OR EXCLUDE ( EXACTKEYWORD , "Meta Analysis" ) OR EXCLUDE ( EXACTKEYWORD , "Adolescent" ) OR EXCLUDE ( EXACTKEYWORD , "Heart Rehabilitation" ) OR EXCLUDE ( EXACTKEYWORD , "Spinal Cord Injury" ) OR EXCLUDE ( EXACTKEYWORD , "Cardiac Rehabilitation" ) OR EXCLUDE ( EXACTKEYWORD , "Cerebral Palsy" ) OR EXCLUDE ( EXACTKEYWORD , "Meta-analysis" ) ) AND ( EXCLUDE ( SUBJAREA , "DENT" ) OR EXCLUDE ( SUBJAREA , "VETE" ) OR EXCLUDE ( SUBJAREA , "EART" ) OR EXCLUDE ( SUBJAREA , "ECON" ) OR EXCLUDE ( SUBJAREA , "ENER" ) OR EXCLUDE ( SUBJAREA , "BUSI" ) OR EXCLUDE ( SUBJAREA , "ENVI" ) OR EXCLUDE ( SUBJAREA , "ARTS" ) OR EXCLUDE ( SUBJAREA , "PHYS" ) OR EXCLUDE ( SUBJAREA , "ENGI" ) OR EXCLUDE ( SUBJAREA , "COMP" ) ) AND ( LIMIT-TO ( DOCTYPE , "ar" ) OR LIMIT-TO ( DOCTYPE , "re" ) ) AND ( LIMIT-TO ( SRCTYPE , "j" ) )
- Search strategy of Web of science
  - (ALL=(Stroke OR Traumatic brain injury OR Neurodegenerative diseases OR Cognitive Dysfunctions) OR TS=(Stroke OR Traumatic brain injury OR Neurodegenerative diseases OR Cognitive Dysfunctions)) AND (ALL=(Telemedicine OR Telerehabilitation) OR TS=(Telemedicine OR Telerehabilitation)) AND (ALL=(Cognition OR Memory OR Attention OR Executive Function OR Quality of Life OR Activities of Daily Living) OR TS=(Cognition OR Memory OR Attention OR Executive Function OR Quality of Life OR Activities of Daily Living))
- Search strategy of KMbase
  - ([ALL=Stroke] OR [ALL=Traumatic brain injury] OR [ALL=Neurodegenerative diseases] OR [ALL=Cognitive Dysfunctions]) AND ([ALL=Telemedicine] OR [ALL=Telerehabilitation]) AND ([ALL=Cognition] OR [ALL=Memory] OR [ALL=Attention] OR [ALL=Executive Function] OR [ALL=Quality of Life] OR [ALL=Activities of Daily Living])
